# Adherence and Health Problems in Thai Travellers Living with HIV

**DOI:** 10.3390/tropicalmed7070128

**Published:** 2022-07-09

**Authors:** Krit Madsalae, Thundon Ngamprasertchai, Saranath Lawpoolsri, Rujipas Sirijatuphat, Winai Ratanasuwan, Watcharapong Piyaphanee, Punnee Pitisuttithum

**Affiliations:** 1 Department of Clinical Tropical Medicine, Faculty of Tropical Medicine, Mahidol University, Bangkok 10400, Thailand; krit.mad@cra.ac.th (K.M.); watcharapong.piy@mahidol.ac.th (W.P.); punnee.pit@mahidol.ac.th (P.P.); 2 Department of Medicine, Chulabhorn Hospital, Chulabhorn Royal Academy, Bangkok 10210, Thailand; 3 Department of Tropical Hygiene, Faculty of Tropical Medicine, Mahidol University, Bangkok 10400, Thailand; saranath.law@mahidol.ac.th; 4 Department of Medicine, Faculty of Medicine Siriraj Hospital, Mahidol University, Bangkok 10700, Thailand; rujipas.sir@mahidol.ac.th; 5 Department of Preventive and Social Medicine, Faculty of Medicine Siriraj Hospital, Mahidol University, Bangkok 10700, Thailand; winai.rat@mahidol.ac.th

**Keywords:** antiretroviral therapy (ART), immunocompromised travellers, HIV travellers, Thai, traveller, adherence and health problems

## Abstract

It is important to focus on adherence to antiretroviral therapy (ART) and health problems of travellers living with HIV (TLWHIV) during travel. This study was conducted to investigate factors related to adherence and health problems among TLWHIV. This multicentre, cross-sectional observational study was conducted among TLWHIV in university hospitals from August 2019 to July 2020. Factors associated with adherence to ART were evaluated using a logistic regression model. Health problems and risk exposure were also examined among participants during travel. Of 321 TLWHIV, 20 (6.23%) showed moderate-to-poor adherence, among whom 3 (15%) had viral rebound after travelling. Travellers frequently missed ART during the first 3 days of their trip. International destination was associated with moderate-to-poor adherence. In total, 237 (73.8%) travellers reported health problems during travel, among whom 36 required medical attention. Sexual or sharp exposure was found in <5% of travellers during travel. Approximately 95% of Thai TLWHIV had good ART adherence. International destination was the major factor determining adherence. TLWHIV should be encouraged to seek pretravel consultation. Healthcare providers should discuss health risk prevention and teach about ART dosing during travel to enhance adherence and minimise toxicity.

## 1. Introduction

Thailand has the highest prevalence of HIV, accounting for 1% of adults, in Southeast Asia [1]. People living with HIV (PLWHIV) tend to live longer and have a better quality of life because of accessibility to effective antiretroviral therapy (ART). Good adherence to ART influences viral suppression, which increases life expectancy. Before the COVID-19 pandemic, tourism was in high demand among Thai and international travellers. As of 2017, there were approximately 9 million Thai international travellers per year [2]. At the same time, as PLWHIV have no limitations on travel, there has been an increase in the number of travellers living with HIV (TLWHIV) [3]. Approximately 46% of HIV-infected people had travelled within a 5-year period [4,5]. TLWHIV are less adherent to ART during travel [6,7]. It has been observed that >60% of travellers reported missing at least one dose of ART, for which the primary reason was being busy and forgetting [7]. Particularly, international travel affects adherence to the timing of ART administration because of the different time zones. Inappropriate ART administration during travel can increase the risk for drug resistance and drug toxicity [8]. Previous studies have shown that most of the TLWHIV generally travel to tropical countries [3,9,10]. Hence, they are exposed to enteric and vector-borne diseases [10]. Moreover, unprotected sex and sharp exposure contribute to blood-borne or body fluid-borne infections (i.e., sexually transmitted infections (STIs)). Morbidities due to STIs should be of particular concern in male travellers and travellers visiting friends and relatives (VFRs) [11]. TLWHIV are likely to have health problems compared with healthy travellers. It has been reported that approximately one-fifth of TLWHIV had an illness that required medical attention during travel [4]. TLWHIV with a CD4 count of <500 cells/mm^3^ were found to have a higher rate of travel-related diseases than did healthy travellers [9].

Pretravel consultation is mandatory to minimise health problems in TLWHIV. Medication planning, risk assessment, and prevention were required to provide for travelers. However, only one-fifth of TLWHIV undergo pretravel preparation before international travel [4]. Healthcare personnel can provide an effective pretravel consultation when they become aware of the factors determining adherence in TLWHIV. This study was conducted to determine ART adherence and explore the factors related to ART adherence in Thai TLWHIV. A second objective was to determine health problems among these travellers.

## 2. Materials and Methods

### 2.1. Study Design, Setting, and Participants

This multicentre, cross-sectional observational study enrolled HIV-infected Thai patients who visited HIV and travel clinics in Siriraj Hospital, Faculty of Medicine, and the Hospital for Tropical Diseases, Faculty of Tropical Medicine, Mahidol University, from August 2019 to July 2020. Inclusion criteria were HIV-infected patients aged at least 18 years who recently had either overnight international or overnight domestic travel within 3 months. Those with symptomatic HIV, such as being currently diagnosed with opportunistic infections, or who were clinically unstable to travel were excluded. Questionnaire-based data regarding baseline characteristics, travel history, self-administered adherence, risk exposure, and health problems were collected. We piloted our questionnaire with 10 participants and modified wherever appropriate. Plasma HIV viral load and CD4 count were also recorded before and after travel as standard of care. All data were confidential and unidentifiable.

### 2.2. Study Procedures

The questionnaire was developed and tested before use. Cronbach’s alpha was used to test the internal consistency of the questionnaire. Five questions and one visual analogue scale (VAS) concerning ART adherence were included in the self-administered adherence at enrolment. On the basis of previous literature [6,7,12,13], we classified the patients into two groups as follows: those who had reported taking complete prescribed doses and had selected VAS 10/10 at the end of the travel or the day before enrolment were classified as having good adherence; however, if they reported missing a single or more doses during their trip till the day before enrolment, they were classified as having moderate-to-poor adherence. When plasma HIV viral load was detectable without any possible causes, those patients were also classified into the moderate-to-poor adherence group.

### 2.3. Sample Size Calculation and Statistical Analysis

According to previous literature [6,7,13], the rate of nonadherence to ART among TLWHIV was 12–60%. The nonadherence rate among Thai HIV-infected patients without travel history was approximately 30–50% [14,15,16]. We estimated that the nonadherence rate among Thai TLWHIV was around 30% ± 5% with 80% power and 5% type I error (two-sided). Accordingly, 325 participants were required in this study.

The good adherence and moderate-to-poor adherence groups were described using appropriate statistical tests (proportion for binary variables, median, and interquartile range (IQR) for non-normally distributed continuous variables and mean and standard deviation for normally distributed continuous variables). For comparing two groups, a chi-square test was used for categorical variables, and the Mann–Whitney U test was used for non-normally distributed continuous variables. Univariable analysis using simple logistic regression was conducted to determine factors associated with adherence. A multiple logistic regression was used to simultaneously investigate the study factors associated with adherence. All factors with a *p* value of <0.25 in the simple logistic regression were included in the multiple logistic regression model. The type of destination (domestic or international) was selected into the model ir-respective of the *p* value, according to previous literature [4,8] and biological plausibility. The analysis was performed using StataCorp. 2019 (Stata Statistical Software: Release 16. College Station, TX, USA: StataCorp LLC).

## 3. Results

Table 1 shows the baseline characteristics of the 321 Thai TLWHIV recruited in this study. Moderate-to-poor adherence was found in 20 (6.23%) travellers. Only 3 (15%) of these 20 travellers showed viral rebound after travel. A high-number pill burden was more associated with good adherence than was a low-number pill burden (OR 0.27; 95% CI 0.01, 2.63) (Table 2). Most of the travellers had well-controlled HIV and more than half disclosed their status to their family or friends. Seven travellers reported that their status had been disclosed during travel. Disclosure HIV status was associated with good adherence without statistical significance. More than half of the travellers had a CD4 count of >500 cells/mm^3^. Approximately 98% had undetectable viral load before travel. Travellers with a CD4 count of ≤500 cell/mm^3^ at baseline had a fourfold moderate-to-low adherence rate, significantly higher than those with a CD4 count of >500 cell/mm^3^ (OR 4.12; 95% CI 1.43, 11.86; *p* = 0.005). The duration of living with HIV and that of ART were approximately 7 and 6 years, respectively, in the good adherence group. These durations between groups were not associated with adherence. More than 95% of them had no history of HIV resistance. The commonly prescribed ART regimen was once-daily, which was associated with good adherence without statistical significance.

Approximately 80% of them had domestic trips, whereas 20% had international trips. Of 75 international travellers, 69 (92%) had good ART adherence. Approximately 44% of international travellers had higher moderate-to-poor adherence than had domestic travellers. The median of trip duration was 4 days. Approximately 80% had short-term trips of <8 days. Long-term trips tended to develop approximately 34% higher moderate-to-poor adherence than did short-term trips. Only 20% of travellers had a pretravel consultation, and they had a lower moderate-to-poor adherence rate of approximately 15%. Univariable analysis revealed that a CD4 count of >500 cells/mm^3^ (OR 0.24; 95% CI 0.07, 0.75; *p* = 0.005), medication pills of more than four tablets/day (OR 0.27; 95% CI 0.01, 2.63; *p* = 0.216), and taking medication other than ART (OR 0.32; 95% CI 0.01, 2.10; *p* = 0.24) were associated with good adherence. Travel destination was associated with adherence when we simultaneously explored all possible factors in the multivariable analysis (OR 13.08; 95% CI 1.10, 154.76, *p* = 0.041).

Travellers frequently missed their ART dose during the first 3 days of their trip followed by on the plane or in the car (Table 3). Most of them skipped their missed dose. The primary reason for the missing dose was forgetting rather than being busy. Moreover, no one reported ART-related stigma or adverse drug reaction.

Sexual risk exposure among travellers was similar to sharp exposure (Table 4). Almost 4% of them had casual sex with either locals or commercial sex workers. Tattoo or body piercing was the most common source of sharp exposure.

Of the 321 travellers, 237 (73.83%) reported health problems during travel. However, only 36 (11.21%) travellers required medical attention when they had health problems during travel. Most self-treated using either over-the-counter drugs or their own medications. A few were seen by a local physician, and only 3% were admitted to a hospital.

## 4. Discussion

The majority of TLWHIV had well-controlled status and were stable on ART for 6–7 average years. Approximately 98% of their viral load was suppressed. Less than 5 % had histories of drug resistance. This well-controlled HIV status suggested that our study participants were concerned with their health status and willing to follow their doctor’s advice. Most of the previous studies [4,6,7,10] were conducted on migrants or those travelling to African countries that have a high health risk. The common destinations in this study were Thailand (domestic) or international (generally Asia). They were familiar with these destinations. It also involves less time zone-crossing travel. Therefore, our study had better adherence than previous studies. Overall, the adherence rate was high and was unrelated to pills per day. Sexual or sharp risk exposure was also lower than what we expected [4]. Several patients also reported mild symptoms of travel-related illnesses, and approximately 10% required medical attention. If more international travellers had been recruited in the study, we might have observed different results. When we simultaneously explored and adjusted for confounders, the only factor that was strongly associated with moderate-to-poor adherence was international destination. Pretravel consultation and ART dosing or toxicity should be advised to international travellers.

Besides self-administered adherence, we evaluated viral rebound after travel to increase adherence validity. The moderate-to-poor adherence rate in our study of approximately 6% was lower than that reported previously (12–60%). Viral rebound after travel was found in <1% of travellers. These findings were consistent with the situation in Thailand in 2019. Approximately 80% of Thai PLWHIV currently had ART, and >97% had suppressed viral load [17]. Thailand is close to achieving the 90–90–90 target for HIV treatment and prevention.

The primary purpose of travel was tourism, which posed a lower risk for health problems than VFRs. Only one-fifth stated that they travelled to visit friends and relatives. Overall, we observed satisfactory results for adherence and risk exposure. This may be in part because travellers who stayed with their family or friends might be more exposed to infectious agents or contact with local residents than those travelling for tourism [18]. Furthermore, VFR travellers tended to have poor ART adherence due to barriers to accessing healthcare or avoiding local medical healthcare during travel [19].

Once-daily, single-tablet regimens promoted good adherence among PLWHIV on ART [20,21]. Our results have a tendency to support previous findings that travellers with comorbidities requiring medications other than ART had a 46% higher moderate-to-low adherence rate than those who were healthy. However, these findings showed statistical insignificance. Similarly, twice-daily ART regimens were associated with moderate-to-low adherence compared with once-daily regimens, but without statistical significance. However, a high number of pills per day was negatively associated with moderate-to-low adherence rate. We hypothesise that travellers taking more than four tablets/day have more focus on health concerns than those taking fewer than four tablets/day. Therefore, simpler medication doses might not affect adherence rate.

Disclosing HIV status impacts adherence. Travellers who disclosed their status were associated with better social support and lesser discrimination [22]. A longer time since diagnosis was related to disclosure status [22]. More than 50% of Thai TLWHIV disclosed their status to family or friends and were living with HIV for >5 years. We detected better adherence rates compared with previous studies that focused on immigrants with poor adherence [6,7].

Travel destinations, particularly international travel, were related to ART adherence. Travelling across time zones affects the timing of ART administration, which might cause viral resistance and medication-related toxicity [8]. Although international travellers were not related to adherence in the univariable analysis, they showed a significant association with moderate-to-poor adherence after adjustment for confounders. Nevertheless, >90% of international travellers had good adherence, which is better than previous data [4,6,7], because those studies focused on African migrants whose destination was generally located in African countries. In our study, Asia was the common destination (i.e., China or Japan), to which air travel took less time than travelling to Africa.

The pretravel consultation rate in this study was 20%, which was lower than that in previous studies [4,23]. Those who had pretravel consultation may have better adherence than travellers who did not. It is possible that travellers with pretravel consultations tend to be more concerned about or aware of travel-related risks, and hence, they also have good adherence. Physicians, including infectious disease care providers or travel medicine specialists, should emphasise specific aspects of the pretravel consultation in TLWHIV (Table 5). As these populations have a high risk of contracting infectious diseases, they should be advised regarding appropriate immunisations or chemoprophylaxis well in advance of travel. Fortunately, our study’s participants had mostly travelled to Asia. Another major issue is the fear of disclosing HIV status during pretravel consultation, as >50% of HIV-infected individuals are discriminated in Thai culture [17]. Appropriate pretravel consultation is impossible if HIV status is unknown, including CD4 count, ART, and HIV viral load. As an example, immunosuppressed travellers may have contraindications to live vaccines. Malaria prophylaxis can also potentially cause drug–drug interactions with ART. Physicians who care for TLWHIV should be encouraged to provide pretravel consultation, particularly to international travellers, because owing to Thai societal perspectives, they might not reveal their status to other physicians.

The major considerations of ART timing during travel were, first, when and how to administer in case of long-distance flights across time zones and, second, how to adjust medication at a destination with a different time zone [8]. Providers should provide detailed counselling on how to adhere to medications, particularly the first few days of a trip, when doses are more likely to be missed. Lastly, medication toxicity is also a concern, as some travellers take their medication instantly when they notice the missing dose. Forgetfulness was the common reason for missing a dose as reported in the literature.

Our study’s strengths were that we followed up the viral load after travel to strengthen the adherence definition, and we retrieved data from both self-response questionnaires and hospital databases. Regarding our study’s limitations, first, this was a cross-sectional study and data were based on patient recall; however, we minimised bias as we interviewed participants after recent travel. Second, we used convenience sampling. We recruited participants from university hospitals in Bangkok; hence, most of them were well-educated and followed treatment guidance. Multicentre studies including various types of hospitals are needed. In addition, most of the participants were domestic travellers, which led to selection bias. We used a *p* value of <0.25 in simple logistic regression in statistical analysis. Finally, participant recruitment was slow during the COVID-19 pandemic because of travel restrictions.

## 5. Conclusions

Our study showed that approximately 95% of Thai TLWHIV had a good ART adherence rate. Most of the travellers had well-controlled HIV status and were on regular treatment for a long period. Most travel was domestic, short-distance air travel, and to Asia. These findings suggest overall good adherence and low-risk exposure in Thai travellers. International destination was the primary factor determining moderate-to-poor adherence. All TLWHIV should be informed about travel health risk in advance, as pretravel consultation was associated with good adherence. Disclosing HIV status during consultation should be considered, as it results in different clinical practice and reduces stigmatization. Healthcare providers discuss techniques to improve ART adherence, particularly during the first 3 days of the trip in which missing dose is common. Moreover, appropriate missing dose administration should be advised individually to reduce ART toxicity during travel. TLWHIV should be encouraged to seek pretravel consultation. Healthcare providers should discuss health risk prevention and ART education our recommendations.

## Figures and Tables

**Table 1:** Baseline characteristics of Thai travellers living with HIV.

Characteristics	Good Adherence (N = 301) n (%) or Mean (SD^+^)	Moderate-to-Poor Adherence (N = 20) n (%) or Mean (SD)	*p*-Value
**Age (years)**	41.88 (11.1)	40.75 (12.8)	0.702
**Sex** Male Female	189 (62.8) 112 (37.2)	13 (65.0) 7 (35.0)	0.843
**Medication** ART other than * Yes No	108 (35.9) 193 (64.1)	9 (45.0) 11 (55.0)	0.412
**Marital status** Single Married Divorced	183 (60.8) 99 (32.9) 19 (6.3)	12 (60.0) 5 (25.0) 3 (15.0)	0.211
**Education level** Below bachelor Bachelor More than bachelor	146 (48.8) 111 (37.1) 42 (14.1)	10 (50.0) 7 (35.0) 3 (15.0)	0.859
**Purpose of travel** Leisure Business Visiting friends Education Pilgrim Medical tourism	140 (46.5) 60 (19.9) 71 (23.6) 7 (2.3) 8 (2.7) 15 (5.0)	11 (55.0) 5 (25.0) 1 (5.0) 0 2 (10.0) 1 (5.0)	0.461
**Viral rebound after travel**	0 (0.0)	3 (15.0)	-

* ART, antiretroviral therapy; SD^+^, standard deviation.

**Table 2:** Factors associated with moderate-to-poor adherence in Thai travellers living with HIV: univariate and multivariate logistic regression analyses.

Factors	Adherence		Univariate Analysis Crude OR * (95% CI)	Multivariate Analysis Adjusted OR (95% CI)
	Good (N = 301) n (%) or Median (IQR^+^)	Moderate-to-Poor (N = 20) n (%) or Median (IQR)		
Travellers’ factors				
**Comorbidities** Yes No	108 (92.3) 193 (94.6)	9 (7.7) 11 (5.4)	1.46 (0.52, 4.01) 1	
**Medication pills per day** >4 ≤4	31 (96.9) 42 (89.4)	1(3.1) 5(10.6)	0.27 (0.01, 2.63) ^#^ 1	0.44 (0.04, 4.78) 1
**HIV factors**				
**Disclosure** Yes No	173 (94.0) 127 (93.4)	11 (6.0) 9 (6.6)	0.9 (0.33, 2.53) 1	
**ART regimen** Twice-daily Once-daily	9 (90.0) 292 (93.9)	1 (10.0) 19 (6.1)	1.71 (0.04,13.49) 1	
**HIV status** **Viral load pretravel** Not suppressed Suppressed	6 (100.0) 259 (93.8)	0 (0) 17 (6.2)	NA	
**CD4 pretravel** ≤500 >500	113 (89.7) 179 (97.3)	13 (10.3) 5 (2.7)	4.12 (1.43, 11.86) ^#^ 1	8.54 (0.82, 89.28) 1
**Years of living with HIV (year)**	7 (3, 14)	5.5 (2.5, 13.5)	0.98 (0.92, 1.1) 1	
**Years of ART (year)**	6 (3, 10)	4.5 (2.5, 10)	0.97 (0.89, 1.05) 1	
**Travel factors**				
Destination ** International Domestic	69 (92) 232 (94.3)	6 (8) 14 (5.7)	1.44 (0.44, 4.17) 1	13.08 (1.10, 154.76) 1
**Direction** Eastward Westward	43 (93.5) 10 (83.3)	3(6.5) 2(16.7)	0.35 (0.04, 4.8) 1	
**Duration of travel** >7 days ≤7 days	60 (92.3) 241 (94.1)	5 (7.7) 15 (5.9)	1.34 (0.37, 4.07) 1	
**Pretravel counselling** Yes No	68 (94.4) 231 (93.5)	4 (5.6) 16 (6.5)	0.85 (0.2, 2.75) 1	
**Taking medications other than ART during travel** Yes No	43 (97.7) 258 (93.1)	1 (2.3) 19 (6.9)	0.32 (0.01, 2.10) ^#^ 1	5.66 (0.30, 107.98) 1

* OR, odds ratio; IQR^+^, interquartile range; ^#^ statistical significance in univariate analysis. (*p* value < 0.25); ** biological plausibility; NA, not applicable.

**Table 3:** Characteristics of moderate-to-poor adherence among Thai travellers living with HIV.

Characteristics	n (%) (N = 16)
**Timing of missing dose** The first 3 days of trip On plane or in car More than 3 days of trip	10 (62.5) 4 (25) 2 (12.5)
**Dealing with missing dose** Skipped dosing Immediately took medication Double dosing Seek medical attention	9 (56.2) 7 (43.8) 0 (0) 0 (0)
**Reason for missing doses** Forgotten Busy Inconvenient storage Stigma Adverse effect	8 (50) 4 (25) 3 (18.8) 0 (0) 0 (0)

**Table 4:** Risk exposure, health problems, and self-care during travel among all groups of Thai travellers living with HIV.

Risk exposure	n (%) (N = 321)
**Casual sex intercourse** With local resident With commercial sex worker	11 (3.43) 8 (72.73) 3 (27.27)
**Sharp exposure** Tattoo Body piercing Shared razor Injection drug use (illicit)	12 (3.74) 5 (41.67) 5 (41.67) 2 (16.67) 0 (0)
**Health problems**	237 (73.83)
**Respiratory tract symptoms** Upper respiratory tract Lower respiratory tract	69 (21.50) 60 (86.96) 9 (13.04)
**Skin** Itching rash Insect bite Urticaria Sunburn	61 (19) 37 (60.66) 13 (21.31) 9 (14.75) 2 (3.23)
**Neurological symptoms** Light-headedness Headache Vertigo	47 (14.64) 16 (34.04) 12 (25.53) 4 (8.51)
**Gastrointestinal tract symptoms** Diarrhoea Abdominal pain Nausea/vomiting	41 (12.77) 27 (65.85) 8 (19.51) 6 (14.63)
**Fever**	19 (5.92)
**Medical help**	36 (11.21)
Self-treated Seen by local physicians Admitted at hospital or clinic	23 (63.89) 10 (27.78) 3 (8.33)

**Table 5:** Authors’ proposed pretravel consultation recommendations.

Issue	Recommendations
HIV status assessment	
- **Baseline including CD4, viral load, and ART before travel** - **The importance of disclosure**	- Doctors assess whether patients’ HIV status is stable. - Doctors should inform patients that disclosure may contribute to better adherence.
**ART education**	
- **Reminder for dosing during the first three days of travel** - **ART toxicities when repeated dose** - **ART timing when crossing time zone** - **ART storage** - **List of medications** - **Extra medication in case of travel delays**	- As ART are frequently missed during this time. - Since repeated dose may lead to toxicities. - Doctors should concern with half-life of ART. - Doctors should suggest patients for a convenient and appropriate ART storage. - Since drug–drug interactions are common among TLWHIV, doctors need to review medications other than ART. - TLWHIV should prevent missing dose during their trip.
**General advice**	
- **Other health status before travel** - **Safe sex** - **Sharp exposure avoidance** - **Jet lag management** - **Vaccination and medication prophylaxis**	- TLWHIV should contact primary doctors before trip. - Doctors educate patients regarding sexually transmitted infections and methods of prevention. - As tattoos or body piercings are popular among TLWHIV. - Doctors should provide indicated medications. - Doctors should consider indicated medications, such as antimalarial prophylaxis or recommended vaccines.

## Data Availability

Not applicable.
